# Acute cyclooxygenase inhibition does not alter muscle sympathetic nerve activity or forearm vasodilator responsiveness in lean and obese adults

**DOI:** 10.14814/phy2.12079

**Published:** 2014-07-17

**Authors:** Jill N. Barnes, Nisha Charkoudian, Luke J. Matzek, Christopher P. Johnson, Michael J. Joyner, Timothy B. Curry

**Affiliations:** 1 Department of Anesthesiology, Mayo Clinic, Rochester, Minnesota; 2 Thermal and Mountain Medicine Division, U.S. Army Research Institute of Environmental Medicine, Natick, Massachusetts

**Keywords:** Autonomic nervous system, blood pressure, forearm blood flow, inflammation

## Abstract

Obesity is often characterized by chronic inflammation that may contribute to increased cardiovascular risk via sympathoexcitation and decreased vasodilator responsiveness. We hypothesized that obese individuals would have greater indices of inflammation compared with lean controls, and that cyclooxygenase inhibition using ibuprofen would reduce muscle sympathetic nerve activity (MSNA) and increase forearm blood flow in these subjects. We measured MSNA, inflammatory biomarkers (C‐reactive protein [CRP] and Interleukin‐6 [IL‐6]), and forearm vasodilator responses to brachial artery acetylcholine and sodium nitroprusside in 13 men and women (7 lean; 6 obese) on two separate study days: control (CON) and after 800 mg ibuprofen (IBU). CRP (1.7 ± 0.4 vs. 0.6 ± 0.3 mg/L; *P *< 0.05) and IL‐6 (4.1 ± 1.5 vs. 1.0 ± 0.1pg/mL; *P *< 0.05) were higher in the obese group during CON and tended to decrease with IBU (IL‐6: *P *< 0.05; CRP: *P *= 0.14). MSNA was not different between groups during CON (26 ± 4 bursts/100 heart beats (lean) versus 26 ± 4 bursts/100 heart beats (obese); *P *= 0.50) or IBU (25 ± 4 bursts/100 heart beats (lean) versus 30 ± 5 bursts/100 heart beats (obese); *P *= 0.25), and was not altered by IBU. Forearm vasodilator responses were unaffected by IBU in both groups. In summary, an acute dose of ibuprofen did not alter sympathetic nerve activity or forearm blood flow responses in healthy obese individuals, suggesting that the cyclooxygenase pathway is not a major contributor to these variables in this group.

## Abstract (precis)

Obesity is often characterized by chronic inflammation that may contribute to increased cardiovascular risk via sympathoexcitation. However, an acute dose of the cyclooxygenase inhibitor ibuprofen did not alter blood pressure or muscle sympathetic nerve activity in lean and obese humans.

## Introduction

Obesity is accompanied by low‐grade chronic inflammation, which may contribute to the increased cardiovascular risk seen in this condition. For example, recent work suggests that inflammation influences the central mechanisms controlling blood pressure (Zubcevic et al. 2011; de Kloet et al. 2013). Along these lines, inflammatory cytokines such as monocyte chemoattractant protein (MCP‐1) are elevated in the nucleus tractus solitarius (NTS) in spontaneously hypertensive rats (Paton and Waki 2009). Furthermore, experimentally increasing IL‐6 in the NTS unfavorably alters blood pressure control (Takagishi et al. 2010). Similarly, in a model of neurogenic hypertension, microinjection of tumor necrosis factor α (TNF‐α) in the hypothalamic paraventricular nucleus (PVN) causes increases in sympathetic nerve activity, which is absent when the TNF‐α is blocked locally (Bardgett et al. 2014). Because the NTS and PVN are major integrators of central autonomic control, this recent work in animal models suggests that inflammatory cytokines may influence sympathetic neural mechanisms in the circulation.

Obesity is often (Grassi et al. 1995, 2001; Ribeiro et al. 2001; Alvarez et al. 2002; Kuniyoshi et al. 2003) but not always (Narkiewicz et al. 1998; Alvarez et al. 2004; Curry et al. 2013) associated with higher sympathetic nerve activity compared to lean control subjects. This chronic sympathoexcitation may contribute to the increased risk for hypertension seen in obesity (Lambert et al. 2013), and may be linked to the elevated levels of inflammatory mediators. Previous studies in humans evaluating the effect of anti‐inflammatory compounds on acute sympathoexcitation have been inconsistent (Doerzbacher and Ray 2001; Monahan and Ray 2005; Cui et al. 2007, 2008). For example, in healthy humans Cui et al. reported that COX inhibition with ketorolac reduced MSNA responses to fatiguing handgrip exercise (Cui et al. 2007, 2008). In contrast, others have reported no change in MSNA response to handgrip exercise (Doerzbacher and Ray 2001) or phenylephrine infusion (Monahan and Ray 2005) after COX inhibition. However, in obese individuals in whom background levels of inflammation are elevated, cyclooxygenase inhibition might unmask an underlying role for inflammatory mediators in chronically altering sympathetic neural control of the circulation in these subjects. In this context, our primary aim was to evaluate sympathetic nerve activity in otherwise healthy obese individuals, compared with lean subjects, with and without a dose of the cyclooxygenase inhibitor ibuprofen. Based on the existing literature, we hypothesized that acute ibuprofen administration would decrease muscle sympathetic nerve activity (MSNA) in our obese subjects, with no effect in the lean subjects.

Obese adults have decreased endothelium‐dependent vasodilation in the forearm (Van Guilder et al. 2006), which could also contribute to increased risk of hypertension in this group. In people with essential hypertension, decreased forearm vasodilation may be related to an increase in endothelium‐derived vasoconstrictor prostaglandins (Taddei et al. 1993, 1997). If the same is true in normotensive people with obesity, cyclooxygenase inhibition may augment vasodilation in this group. Therefore, a secondary aim was to test the hypothesis that cyclooxygenase inhibition would augment endothelium‐dependent forearm vasodilator responses in obese adults.

## Methods

### Participants

A total of 13 adults, aged 18–40 years, gave written consent and participated in the study. All subjects were healthy, as determined by a review of medical history and brief physical exam, nonsmoking, and normotensive. None of the subjects were taking cardiovascular acting medications or had a history of cardiovascular disease or other chronic diseases. Subjects with hypertension, as determined by repeated seated brachial cuff pressure measured during the screening visit, were excluded. Subjects were considered lean if they had a body mass index (BMI) <28 kg/m^2^ and obese if they had a BMI between 35 and 45 kg/m^2^. Young women were studied in the early follicular phase of the menstrual cycle or the low hormone phase of oral contraceptive use to minimize the effects of the reproductive hormones on autonomic or cardiovascular function (Minson et al. 2000). All young women were asked to complete a pregnancy test within 48 h of the study day to ensure that they were not pregnant during study procedures. All procedures were reviewed and approved by the Institutional Review Board at Mayo Clinic and were conducted according to the Declaration of Helsinki.

### Experimental procedures

All of the studies were performed in the Mayo Clinic Clinical Research Unit (CRU) where the ambient temperature was controlled between 22 and 24°C. Subjects arrived at the laboratory after an overnight fast and at least 24 h without caffeine or vigorous exercise. On arrival, subjects rested in the supine position during instrumentation. After local anesthesia with 2% lidocaine, a 5‐cm, 20‐gauge arterial catheter was placed in the brachial artery of the nondominant arm under ultrasound guidance, using aseptic technique. The catheter was connected to a pressure transducer, which was positioned at the level of the heart and interfaced with a personal computer to monitor arterial pressure. A 3‐lead ECG was used for continuous recordings of heart rate (HR). Arterial blood was drawn for the measurement of blood lipoproteins levels, C‐reactive protein (CRP), and IL‐6. High sensitivity CRP was measured using Roche chemistry analyzer (Roche Diagnostics, Indianapolis, IN; lowest detection 0.03 mg/dL). IL‐6 was measured using immunoassay (R&D Systems, Minneapolis, MN; lowest detection 0.31 pg/mL).

Multiunit MSNA was measured from the right peroneal nerve at the fibular head using insulated tungsten microelectrodes. A muscle sympathetic fascicle was identified when taps on the muscle belly or passive muscle stretch evoked mechanoreceptive impulses, and no afferent neural response was evoked by skin stimuli (Sundlof and Wallin 1977, 1978). The recorded signal was amplified 80,000‐fold, band pass filtered (700–2000 Hz), rectified, and integrated (resistance‐capacitance integrator circuit, time constant 0.1 s) by a nerve traffic analyzer (662C‐4 Nerve Traffic Analysis System, University of Iowa, Iowa City, IA). ECG, arterial pressure, and MSNA data were simultaneously recorded at 250 Hz (WinDaq, DATAQ Instruments, Akron, OH). Sympathetic bursts in the integrated neurogram were identified in the recorded data using an automated analysis program (Kienbaum et al. 2001) that assigns each sympathetic burst to the appropriate cardiac cycle by compensating for latency. The automated analysis was then reviewed by study personnel blinded to the specifics of the study day and corrected manually. In addition to the cardiovascular and neurophysiological criteria included in the automated program (Kienbaum et al. 2001), a minimum of a 3:1 signal to noise ratio was used for burst confirmation. MSNA is reported as bursts/minute (burst frequency) and bursts/100 heartbeats (burst incidence).

Hemodynamic data were analyzed offline using data acquisition software (WinDaq, DATAQ Instruments, Akron, OH). Beat‐to‐beat stroke volume was calculated from the brachial arterial waveform as previously described (Hart et al. 2011), using Modelflow analysis. Briefly, aortic waveform was computed based on nonlinear pressure–volume, pressure–compliance, and pressure–characteristic impedance equations, incorporating age, sex, height, and body mass (Wesseling et al. 1993). Cardiac output was calculated as stroke volume × heart rate and TPR was calculated as MAP/cardiac output.

Forearm blood flow (FBF) was measured using mercury‐in‐silastic strain gauge plethysmography (Joyner et al. 2001). Briefly, an occlusion pressure cuff was placed around the wrist and inflated to suprasystolic levels (220 mmHg) to arrest the circulation of the hand, and a venous occlusion cuff was placed on the upper arm and rapidly inflated to 50 mmHg every 7.5 s, yielding one blood flow every 15.0 s. FBF was expressed as milliliters per 100 ml of tissue per minute. Forearm vascular conductance was calculated from FBF and MAP (FVC = FBF/MAP) and expressed as milliliters per 100 mL of tissue per 100 mmHg.

### Brachial artery drug infusions

All study drugs were administered via the brachial artery catheter at total rates of 2–4 mL/min depending on the individual subject's forearm volume (as determined by water displacement). To evaluate endothelium‐dependent vasodilation, acetylcholine (ACH) was administered for 2 min each at 2, 4, and 8 *μ*g/dL/min, respectively. To evaluate endothelium‐independent vasodilation, sodium nitroprusside (NTP) was administered for 2 min each at 0.5, 1.0, and 2.0 *μ*g/dL/min, respectively.

### Drug administration

On one of the study days, subjects received 800 mg oral ibuprofen (IBU) in pill form approximately 90 min prior to the start of data collection. The control (CON) and IBU days were randomized and separated by at least 1 month.

### Data analysis and statistics

Baseline MSNA for CON and IBU study days was calculated as the average of 5 min of resting data prior to the start of FBF dose–response trials. Similarly, resting HR (from ECG) and arterial pressure data were calculated as 5‐min averages. FBF data were averaged during the last minute of each of the 2‐min stages (baseline and each drug dose). FBF was divided by mean arterial pressure to derive an index of forearm vascular conductance (FVC).

Data were analyzed statistically using commercially available software (Sigma Stat 12; SPSS Inc., Chicago, IL). Group data are expressed as mean ± SEM. A one‐way ANOVA was used to determine group differences in subject characteristics. Repeated measures ANOVA was used to compare MSNA, hemodynamics, and forearm blood flow responses during ibuprofen (IBU) and control conditions (CON). A multifactorial ANOVA was used to examine whether neural and cardiovascular variables were different in response to ibuprofen within and between lean and obese groups, with a Bonferroni adjustment for pairwise comparisons. The association between MSNA and inflammatory cytokines was determined using Spearman's rho. The *α*‐level was set at 0.05.

## Results

Subject characteristics are listed in Table 1. Obese adults had higher body mass, BMI, and fasting insulin compared with lean adults. The range of BMI for the lean adults was 20–26 kg/m^2^ and 35–40 kg/m^2^ in the obese adults.

**Table 1.** Subject characteristics

	Lean	Obese
M/F, *n*	4/3	5/1
Age, years	25 ± 3	30 ± 3
Height, cm	178 ± 4	178 ± 3
Body mass, kg	74 ± 4	116 ± 6*
Body mass index, kg/m^2^	23.3 ± 0.8	36.5 ± 0.9*
Fasting glucose, mg/dL	93 ± 1	99 ± 5
Fasting insulin, *μ*IU/mL	7 ± 1	37 ± 14*

Mean ± SEM.

* *P *< 0.01 versus Lean.

### Indices of inflammation

**Figure 1.**
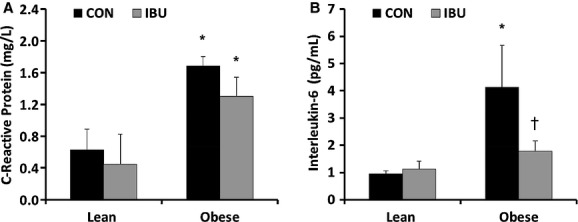
C‐reactive protein (A) and interleukin‐6 (B) in lean and obese adults during control (CON) (black bars) and ibuprofen (IBU) (grey bars). Data are mean ± SE, *P < 0.05 compared to lean adults, ^†^*P *< 0.05 compared to CON.

C‐reactive protein (CRP) was higher in obese adults compared with lean adults (Fig. 1) and there was a slight, but nonsignificant, decrease in CRP with IBU (*P *= 0.14). IL‐6 was elevated in obese adults during CON (Fig. 1), but was not different from lean adults during IBU (Fig. 1). Importantly, IL‐6 was lower during IBU in obese adults only, indicating a reduction in inflammatory markers.

### Hemodynamic and neural variables

**Figure 2.**
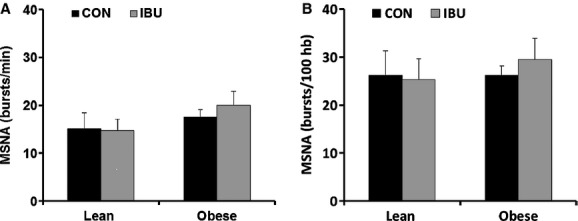
Muscle sympathetic nerve activity (MSNA) burst frequency (A) and MSNA burst incidence (B) in lean and obese adults during control (CON) (black bars) and ibuprofen (IBU) (grey bars). Data are mean ± SE.

**Figure 3.**
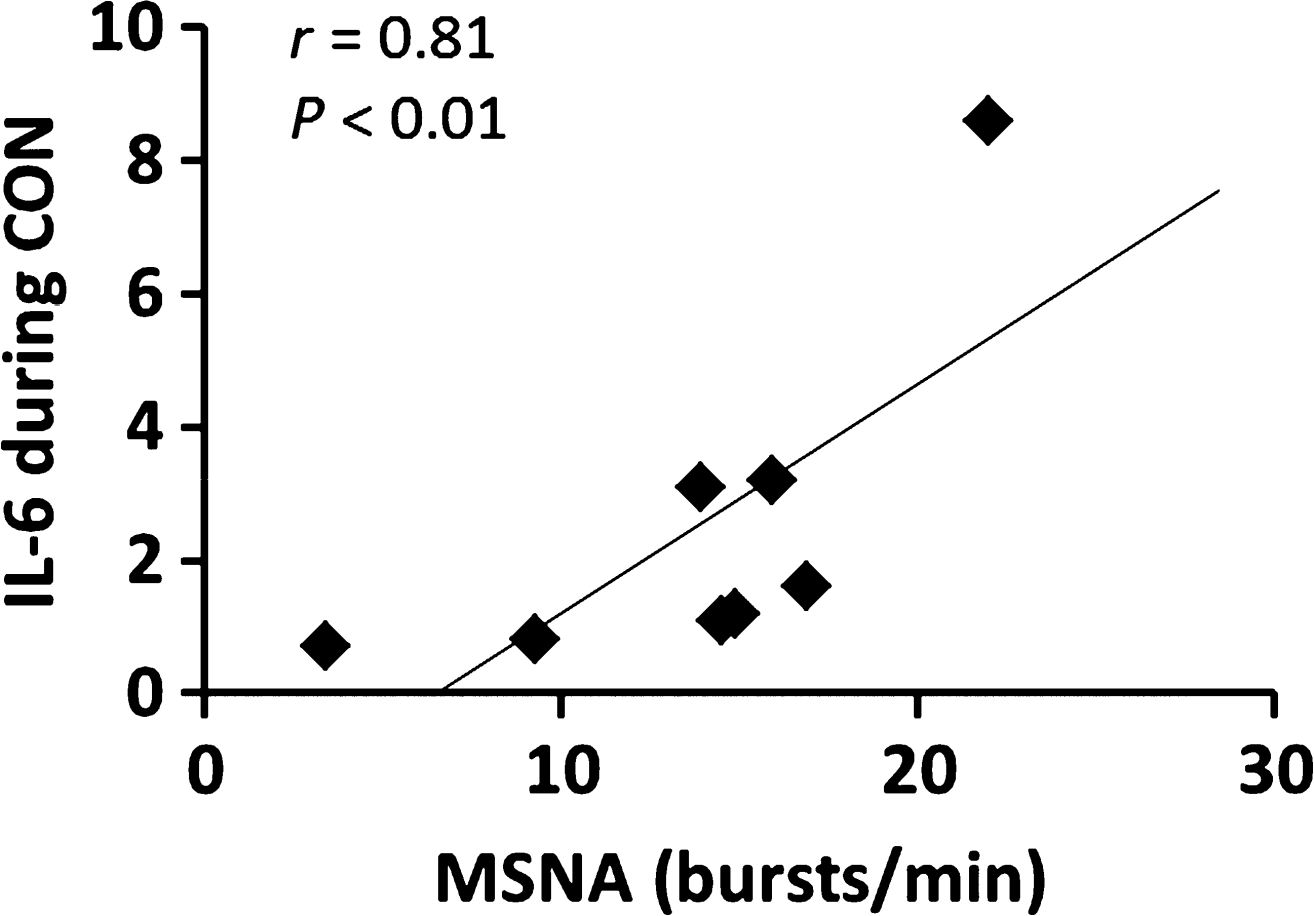
The association between muscle sympathetic nerve activity (MSNA) burst incidence and circulating concentrations of interleukin‐6 (IL‐6) during control (CON). The positive association between MSNA and IL‐6 was significant only during the CON trial.

Although none of the participants had history of hypertension, mean arterial blood pressure (MAP) was higher in obese adults compared with lean adults during both CON (107 ± 4 vs. 93 ± 4 mmHg; *P *< 0.01) and IBU (103 ± 4 vs. 90 ± 3 mmHg; *P *< 0.01) and there were no significant changes between CON and IBU study days (Table 2). Heart rate at rest and cardiac output were higher in obese adults compared with lean adults in both the CON and IBU study days. There were no group differences and no effect of ibuprofen administration on total peripheral resistance. MSNA burst frequency (bursts/min) and burst incidence (bursts/100 heart beats) were not different between groups during CON or IBU, and were not altered by ibuprofen (Fig. 2). Interestingly, there was a significant positive association between IL‐6 and MSNA in the combined group of subjects during CON (Fig. 3).

**Table 2.** Hemodynamic responses to ibuprofen

	Lean		Obese	
	CON	IBU	CON	IBU
Heart rate, bpm	57 ± 2	59 ± 2	67 ± 2*	68 ± 3*
Systolic blood pressure, mmHg	130 ± 3	128 ± 3	142 ± 7	140 ± 6
Diastolic blood pressure, mmHg	71 ± 2	70 ± 2	86 ± 6	82 ± 2
Mean arterial pressure, mmHg	92 ± 2	90 ± 2	108 ± 6*	103 ± 3*
Cardiac output, L/min	4.5 ± 0.3	4.6 ± 0.4	6.1 ± 0.4*	6.4 ± 0.3*
Total peripheral resistance, mmHg/L/min	21 ± 2	20 ± 2	18 ± 3	16 ± 1

CON, control; IBU, ibuprofen.

Mean ± SEM.

* *P *< 0.05 versus Lean.

### Forearm vascular responses

**Figure 4.**
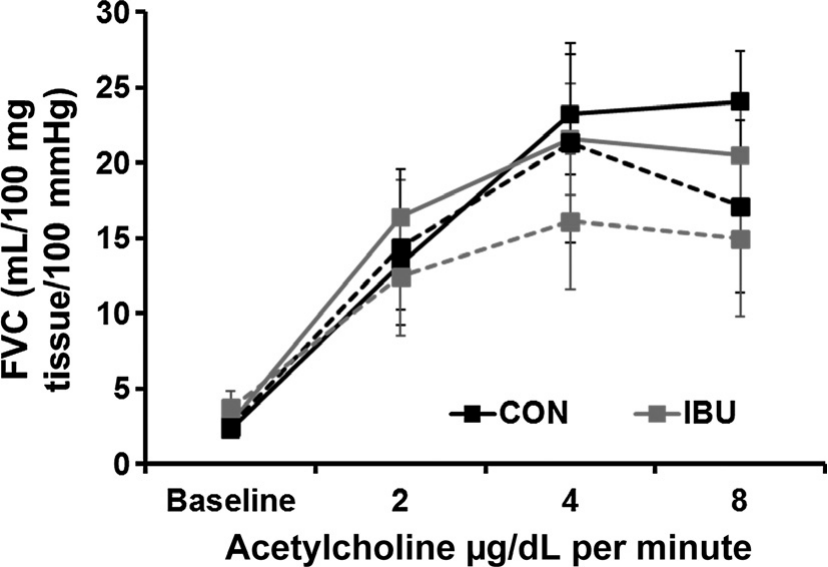
Endothelium‐dependent vasodilation was measured using forearm vascular conductance (FVC) at baseline, and in response to three doses of acetylcholine. There were no differences between lean (solid lines) and obese (dotted lines) during control (CON) (black) or ibuprofen (IBU) (grey). There were also no differences in FVC during nitroprusside infusion (data not shown).

Forearm vascular conductance (FVC) at baseline and during increasing doses of acetylcholine are shown in Figure 4 for both groups. There were no statistical differences between groups or conditions. In the interest of being concise, we only present selected data here and in Figure 4. There were no differences in FVC at baseline between lean and obese adults (2.3 ± 0.4 vs. 2.5 ± 0.3 mL/100 mg tissue/100 mmHg; *P *> 0.05). In addition, peak FVC responses to both acetylcholine (24.1 ± 3.4 vs. 21.3 ± 6.6 mL/100 mg tissue/100 mmHg; *P *> 0.05) and nitroprusside (17.6 ± 3.4 vs. 17.1 ± 4.1mL/100 mg tissue/100 mmHg; *P *> 0.05) were similar in both lean and obese adults, and not altered by cyclooxygenase inhibition (data not shown; *P *> 0.05).

## Discussion

The major new finding of this study was that, despite higher indices of chronic inflammation in our obese subjects, acute oral IBU administration did not alter sympathetic nerve activity in this group. Similarly, IBU did not alter MSNA in our control group of lean adults, which is consistent with previous observations of a lack of effect of cyclooxygenase inhibition on MSNA in healthy controls (Doerzbacher and Ray 2001; Monahan and Ray 2005). Forearm vasodilator responses were similarly unaffected by IBU administration in this study. Taken together, these findings suggest that in healthy obese subjects, the cyclooxygenase pathway has a minimal role in the control of sympathetic nerve activity and vascular endothelial function.

Evidence from animal models suggests that pro‐inflammatory molecules may activate central nuclei responsible for control of sympathetic neural activity in hypertension (Waki et al. 2008; Paton and Waki 2009). For example, gene expression of pro‐inflammatory cytokines was elevated in the nucleus tractus solitarius of spontaneously hypertensive rats (SHR), suggesting that these mediators may contribute to sympathetic activation in this model of neurogenic hypertension (Waki et al. 2008). Obesity is often associated with increased markers of inflammation, in part because adipocytes themselves release adipokines, including IL‐6, TNF‐α, and CRP (Trayhurn and Wood 2004; Smith and Minson 2012; Proenca et al. 2014). These adipokines have been hypothesized to be associated with the sympathetic overactivity and hypertension seen in some forms of obesity (Smith and Minson 2012). In this study, we reasoned that a potential pro‐inflammatory state in obese subjects would be associated with similar alterations in central neural control as those seen in the SHR model (Waki et al. 2008). Our obese subjects demonstrated significantly higher CRP and IL‐6 levels compared to our lean control group.

In the context of this study, ibuprofen crosses the blood–brain barrier and can therefore have anti‐inflammatory effects both centrally and peripherally (Parepally et al. 2006). We were not able to specifically evaluate central (brain) anti‐inflammatory influences. However, a single 800 mg dose of IBU did not alter MSNA in either group. Thus, our results suggest that if low‐level inflammation does contribute to the level of sympathetic nerve activity in healthy obese adults, its mechanism does not include the cyclooxygenase pathway. It is possible that other noncyclooxygenase mechanisms contribute to the prevailing level of sympathetic nerve activity in obese adults. Although there was no influence of IBU on MSNA in this study, the decrease in IL‐6 with IBU suggests that this dose was anti‐inflammatory in our subjects.

In this study, the lack of difference in baseline MSNA between our control and obese groups may seem surprising, given the evidence in the literature supporting chronic sympathoexcitation in obesity (Grassi et al. 1995; Ribeiro et al. 2001; Alvarez et al. 2002; Kuniyoshi et al. 2003). However, reports of sympathetic nervous system activation in obesity are inconsistent (Young and Macdonald 1992), and we and others have noted extensive interindividual variability in the amount of resting MSNA in humans (Sundlof and Wallin 1977; Charkoudian et al. 2005; Hart et al. 2011).

Our recent experience with obese subjects suggests that a subset of obese individuals may not have higher MSNA than their lean counterparts (Curry et al. 2013). The reasons for this variability among obese subjects likely relate to the complexity of the interaction between body fatness and MSNA at rest. For example, MSNA is much more strongly related to abdominal visceral adiposity than it is to subcutaneous adiposity or total fat mass (Alvarez et al. 2002). Indeed, in a group of men with primarily subcutaneous obesity, MSNA was not different from a group of lean men (Alvarez et al. 2004). A limitation of this study was that we did not evaluate the distribution of body fat in our present subjects. However, it may be that our subjects had less visceral, and more subcutaneous, distribution of body fat, thus contributing to a lower relative level of MSNA compared to other groups of obese individuals. Additionally, our relatively small sample size of obese subjects (*n *= 6) may have limited our ability to detect differences.

The above considerations may also have implications for our understanding of the interactions among exercise training, adiposity, and sympathetic control of the circulation. Alvarez and colleagues reported that endurance exercise training was associated with *higher* MSNA when trained men were compared to sedentary men with similar levels of abdominal adiposity (Alvarez et al. 2005). In comparisons where abdominal adiposity was higher in sedentary, the exercise trained and sedentary groups did not have different MSNA (Alvarez et al. 2005). Thus, exercise training status and body fatness have interactive influences that can confound interpretation because both can (separately) increase MSNA. In this study, none of our subjects self‐reported that they did any more than recreational physical activity; however, we did not specifically control for this variable (beyond self‐report), which may have contributed to some of the variability observed.

The primary aim of this study was to evaluate whether ibuprofen administration would alter MSNA, and we found no difference in MSNA between the control and ibuprofen study days. One limitation of this study is the small sample size that may have prevented us from finding statistical differences. In addition, because we recruited healthy obese adults, it is possible that the overall inflammatory burden was not sufficiently high to alter neural and hemodynamic variables. However, importantly, the obese group in this study did show significantly elevated levels of inflammatory biomarkers, including CRP and IL‐6. While IL‐6 was lower after ibuprofen in obese adults, there was no effect on CRP. Additionally, IL‐6 concentrations were positively associated with MSNA across all subjects in the CON trial, reinforcing the idea of a link between inflammation and sympathetic activity (Fig. 3). Therefore, our findings suggest that the cyclooxygenase pathway is not involved in any potential link between chronic low‐level inflammation and sympathetic neural mechanisms in obese humans. It is possible, however, that longer term COX inhibition would have more pronounced influences compared to a single dose of IBU. This is an important direction for future work.

A secondary goal of this study was to measure forearm blood flow responses to acetylcholine and nitroprusside to evaluate endothelium‐dependent and ‐independent vasodilation, respectively. Earlier work from Taddei and colleagues showed that FBF responses to ACH were blunted in essential hypertension (Taddei et al. 1993, 1997), and that cyclooxygenase inhibition with indomethacin augmented the ACH responses (Taddei et al. 1993, 1997). They concluded that endothelium‐derived vasoconstrictor factors, in particular vasoconstrictor prostanoids, were responsible in part for the blunted vasodilation seen in their hypertensive subjects. In designing this study, we reasoned that if such endothelium‐derived vasoconstrictor factors were present in our obese group, then an acute dose of IBU might augment vasodilation to ACH in this group. We did not observe blunted vasodilation in our obese group nor did IBU have an effect on the vasodilation in either group. This may be related to the fact that our obese subjects were otherwise healthy, as obese subjects with additional cardiovascular risk factors or metabolic syndrome would be more likely to show impaired vasodilation (Limberg et al. 2012) and pathophysiological changes in endothelium‐dependent vasodilation.

## Summary and Conclusions

In summary, we report that an acute dose of 800 mg ibuprofen did not alter sympathetic nerve activity or vascular endothelial function in healthy lean and obese adults, in spite of elevated markers of systemic inflammation in the obese group. Our findings suggest that, in spite of subclinical elevations in systemic inflammatory biomarkers in healthy obese adults, the cyclooxygenase pathway did not play a significant role in the mechanisms controlling vascular sympathetic nerve activity or forearm vasodilation. We are careful to note in this context that additional cardiovascular risk factors (e.g., metabolic syndrome) may be associated with higher levels of systemic inflammation and a different role for these pathways in modulating sympathetic control of the circulation.

## Acknowledgments

The authors would like to thank Shelly Roberts, Jessica Sawyer, Casey Hines, and Shirley Kingsley‐Berg for their continued assistance throughout the project. Its contents are solely the responsibility of the authors and do not necessarily represent the official views of the National Institutes of Health. The views, opinions, and/or findings contained in this article are those of the authors and should not be construed as an official Department of the Army position, or decision, unless so designated by other official documentation. Approved for public release; distribution unlimited.

## Conflict of Interest

None declared.
